# Ten-year outcomes after percutaneous coronary intervention versus coronary artery bypass grafting for multivessel or left main coronary artery disease: a systematic review and meta-analysis

**DOI:** 10.1186/s13019-023-02101-y

**Published:** 2023-02-02

**Authors:** Shitao Feng, Mingli Li, Jiayue Fei, Anqin Dong, Wenli Zhang, Yanhua Fu, Yang Zhao

**Affiliations:** Department of Geriatrics, The Fifth Affiliated Hospital of Zhengzhou University, 3 Kang fu Qian Street, Er Qi District, Zhengzhou, 450052 Henan People’s Republic of China

**Keywords:** Percutaneous coronary intervention, Coronary artery bypass grafting, Meta-analysis

## Abstract

**Background:**

Short-term and long-term comparative outcomes after percutaneous coronary intervention (PCI) and coronary artery bypass grafting (CABG) for multivessel coronary artery (MVCA) or left main coronary artery (LMCA) disease are highly debated.

**Goals:**

We performed a meta-analysis to evaluate the difference between PCI and CABG for the treatment of patients with MVCA or LMCA in long-term follow-up.

**Methods:**

Literatures were searched in PubMed, EMBASE and The Cochrane Library from January 1, 2000 to January 1, 2021, including RCTs and observational studies (OSs). The primary outcome was all-cause mortality at 10 years follow-up, and the secondary outcomes included cardiac mortality, repeated revascularization, myocardial infarction, and stroke.

**Results:**

A total of 5 RCTs reporting data from 3013 participants and 4 OSs of 5608 participants were included for analysis. There was no significant difference between PCI and CABG in all-cause mortality (Odds Ratio (OR) 1.03 [95% confidence interval (CI) 0.89 to 1.19]), whereas PCI was associated with higher cardiac mortality (OR 0.76 [95% CI 0.65 to 0.90]) and repeated revascularization rate comparing to CABG (OR 1.77 [95% CI 1.08 to 2.89]; I^2^ = 94.61%). The difference between PCI and CABG in repeated revascularization in either RCTs or OSs, in myocardial infarction in either RCTs or OSs were not significant. In OSs, stroke rate in PCI group was lower than those in CABG, but not in RCTs. There was a significant increase of stroke rate in CABG comparing to PCI (OR 0.65 [95% CI 0.53 to 0.80]; I^2^ = 0.00%). No significant difference between PCI and CABG in myocardial infarction was not observed (OR 0.92 [95% CI 0.64 to 1.31]; I^2^ = 57.84%).

**Conclusion:**

Evidence from our study and prior studies suggested the superiority of CABG over PCI in improving 5- but not 10-year survival among patients with MVCA. In the contrast, there was no significant difference between CABG and PCI for treating patients with LMCA in either 5- or 10-year survival rate. More long-term trials are needed to better define differences of outcome between 2 techniques.

**Supplementary Information:**

The online version contains supplementary material available at 10.1186/s13019-023-02101-y.

## Introduction

In the past few decades, several randomized clinical trials (RCTs) have compared percutaneous coronary intervention (PCI) with coronary artery bypass grafting (CABG) in patients with multiple vessels coronary artery disease (MVCA) or left main coronary artery disease (LMCA), as CABG was associated with lower incidence of all-cause death than PCI for patients with LMCA or MVCA in 5-year follow-up [1–4]. However, conflicting results between short-term and long-term comparative outcomes were found in this field. Patients with LMCA treated by CABG did not demonstrate significant difference in the incidence of all-cause death than PCI in the 10-year data [5]. On the contrary, a newly revealed RCT failed to demonstrate the non-inferiority of fractional flow reserve-guided PCI comparing to CABG in 1-year follow-up, triggering a heated discussion with regard to the most optimal treatment between PCI and CABG in treating MVCA or LMCA [6].

The 2018 European Society of Cardiology/European Association for Cardio-Thoracic Surgery Guidelines on myocardial revascularization assigned a Class I recommendation (“is recommended”) to CABG to improve outcomes among patients with LMCA or MVCA [7]. However, the supporting evidences were studies with no more than 5 years follow-up. To our best knowledge, there has been no meta-analysis of long-term (10 years) follow-up performed to compare the safety and efficacy of PCI and CABG in treating MVCA or LMCA. In this study, we comprehensively collected data and evaluated the difference in both RCTs and observational studies (OSs) that have compared PCI and CABG for the treatment of patients with MVCA or LMCA in 10-year follow-up, providing further insights into the comparative advantages of both revascularization techniques.

## Methods

All supporting data in this article are available. Literatures were comprehensively searched by 2 reviewers in PubMed, EMBASE, Cochrane Library (Cochrane Database of Systematic Reviews, Cochrane Central Register of Controlled Trials, Cochrane Methodology) from January 1, 2000, to January 1, 2021. Details of searching algorithm were listed in the Additional file 1. This study was directed by the Preferred Reporting Items for Systematic Reviews and Meta-Analysis guidelines for RCTs [8], along with the Meta-Analysis of Observational Studies in Epidemiology for OSs [9], and was registered at PROSPERO, number CRD42021247485.

We included studies comparing outcomes between PCI and CABG in treating coronary artery disease (CAD) of either MVCA or LMCA in 10 years follow-up. RCTs, prospective and retrospective OSs were all taken into consideration. Detailed selection criteria for inclusion/exclusion were showed in the Additional file 1.

The risk of bias was evaluated by 2 independent reviewers. Cochrane risk of bias assessment was used to evaluate RCT’s publication bias of including selection bias, performance bias, detection bias, attrition bias, reporting bias, and other sources of bias [10]. The quality of OSs was assessed by using the Newcastle-Ottawa Scale [11].

Titles and abstracts of all studies were collected by 2 authors from databases mentioned above. All eligible studies were screened based on the inclusion and exclusion standards in Additional file 1. Divergences were resolved by consensus. Following data were extracted: (1) study features: authors, study design, sample size and quality of studies; (2) baseline information of patients; (3) outcomes: the primary outcomes was all-cause mortality at 10 years follow-up; the secondary outcomes included cardiac mortality, repeated revascularization, myocardial infarction and stroke at 10 years follow-up. Due to the variable definitions of major adverse cardiac and cerebrovascular event (MACCE) in different studies, we did not use MACCE as a measure of outcomes in this study [4, 12].

The Comprehensive Meta-Analysis 3.0 (Biostat, Englewood, NJ) was used to perform the data analysis. Owing to the intrinsic differences between RCTs and OSs, separate analyses of these 2 types of study designs were conducted. Odds ratios (ORs) with 95% confidence interval (CI) were measured and pooled for each outcome. Thanks to the diverse clinical features and methodological differences, a random-effect model was utilized for analyses [13]. We used the Q test and the calculation of I^2^ for the assessment of heterogeneity between studies. Substantial heterogeneity would be considered if P < 0.05 or I^2^ ≥ 50%. Subgroup and sensitivity tests were performed to investigate the source of heterogeneity. Due to the heterogeneity between studies, subgroup and sensitivity tests were performed in the categorization based on lesion location of patient^,^ s baseline (LMCA or MCA) or types of PCI used in the studies(DES or BMS). Difference between subgroups was assessed by z test, and 2-tailed P < 0.05 was regarded as statistically significant.

## Results

**Fig. 1:**
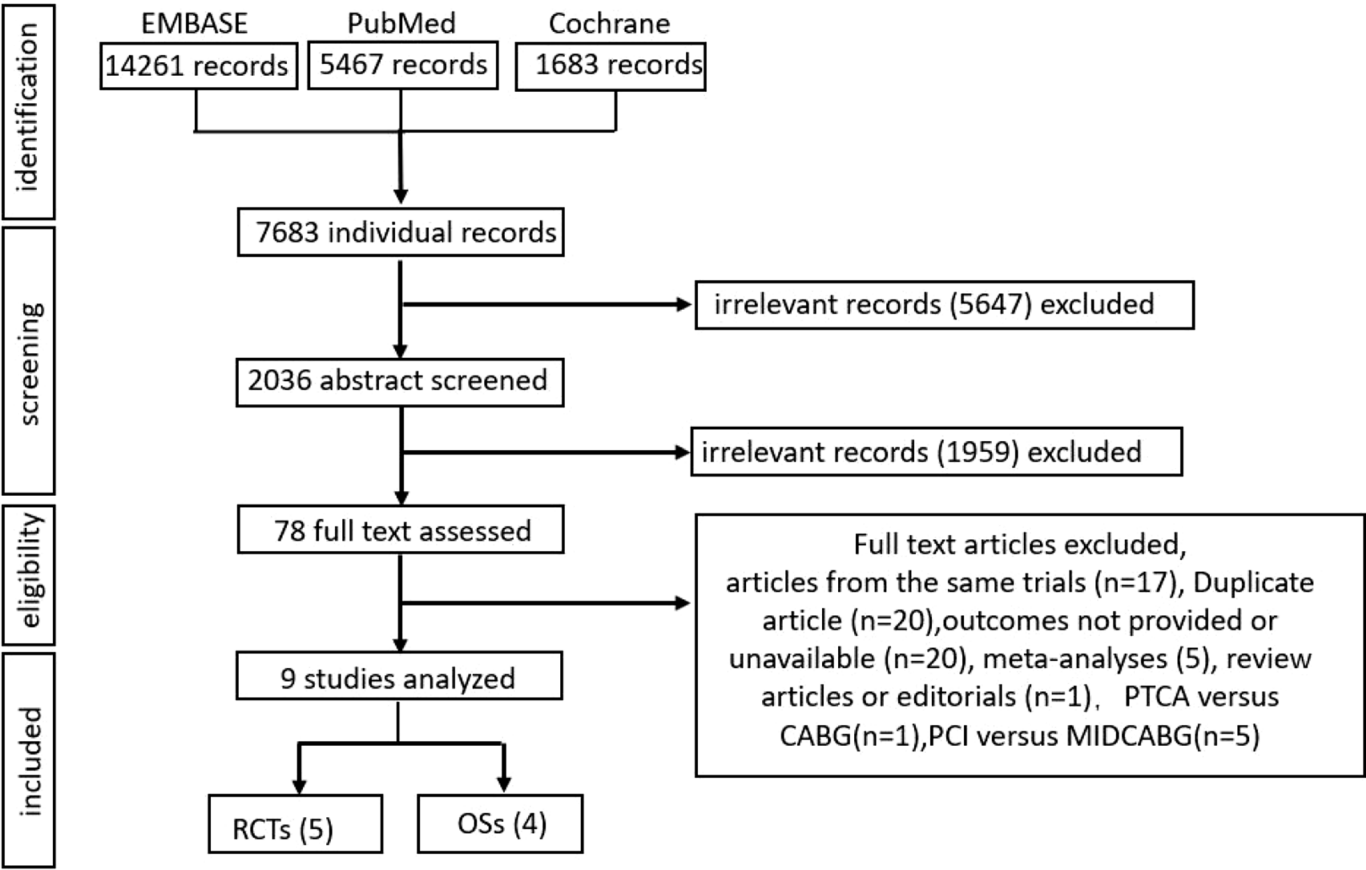
Flow diagram of the research

A total of 5 RCTs [5, 14–17] reporting data from 3013 participants and 4 OSs [18–21] of 5608 enrolled participants were included for analysis. A flowchart indicating selection strategy was showed in Fig. 1 and the baseline information of the participants was showed in Table 1. According to the Cochrane collaboration’s tool, the risk of bias in RCT was rated low (Additional file 1: Table S1). Owing to low comparability, 2 out of 4 studies were calculated as 6 by the Newcastle-Ottawa scale, while the rest studies were above 6 (Additional file 1: Table S2).

**Table 1:** All studies included in the meta-analysis

Study	SYNTAX PCI/CABG	PRECOMBAT PCI/CABG	SOS PCI/CABG	MASS II PCI/CABG	LE MANS PCI/CABG	MAIN-COMPARE PCI/CABG	ASAN-MAIN PCI/CABG	Nystrom2017 PCI/CABG	Yu2020 PCI/CABG
Study design	RCT	RCT	RCT	RCT	RCT	Observational study	Observational study	Observational study	Observational study
Type of lesion	LMCA/MCAD	LMCA	MCAD	MCAD	LMCA	LMCA	LMCA	NR	LMCA
Patients	903/897	300/300	50/50	205/203	52/53	1102/1138	100/250	1863/683	271/201
Age	65.2 (9.7)/65.0 (9.8)	61.8 (10.0)/62.7 (9.5)	54.7 (8.8)/52.7 (9.1)	60 (9)/60 (9)	60.6 (10.5)/61.3 (8.4)	61.3 (11.7)/62.9 (9.4)	55.1 (10.4)/60.7 (9.1)	61.1 (10.5)/57.2 (10.0)	61.7 (10.3)/60.6 (8.8)
Male sex, %	76.0/79.0	76.0/77.0	80.0/78.0	67.0/72.0	60.0/73.0	70.7/72.9	60.0/74.4)	58.9/63.4	74.2 /76.1
BMI, mean	28.1 (4.8)/27.9 (4.5)	24.6 (2.7)/24.5 (3.0)	NR	NR	NR	NR	24.6 (3.1)/24.6 (2.7)	26.0 (3.9)/26.4 (4.3)	NR
Diabetes mellitus, %	26.0/25.0	34.0/30.0	14.0/18.0	23.0/29.0	19.0/17.0	29.7/34.7	21.0 /32.8	100.0/100.0	28.8/28.9
Hypertension, %	69.0/64.0	54.3/51.3	54.0/42.0	61.0/63.0	75.0/70.0	49.5/49.4	23.0/50.0	NR	56.1/50.2
Hyperlipidemia, %	79.0/77.0	42.3/40.0	46.0/50.0	NR	65.0/60.0	28.6/32.6	34.0/46.0	NR	49.8/38.8
smoker, %	18.0/22.0	29.7 /27.7	44.0/40.0	27.0/32.0	NR	25.6/29.8	36.0/27.2	14.5/15.0	48.3/46.2
previous MI ,%	32.0/34.0	4.3/6.7	38.0/36.0	52.0/41.0	36.0/32.0	8.1/11.6	14.0/16.0	47.6/54.3	17.3/26.9
Previous CVA ,%	4.0/5.0	NR	NR	NR	NR	7.1/7.3	4.0./16.0	9.6/6.6	2.2/14.4
LVEF, %	NR	61.7 ± 8.3 /60.6 ± 8.5	NR	67.0 ± 8.0 /67.0 ± 9.0	53.5 ± 10.7/ 53.7 ± 6.7	60.6 ± 10.8 /57.2 ± 11.9	60.3 ± 9.1 /56.8 ± 11.9	NR	64.0 ± 8.1 /62.0.±11.1
LMCA only, %	42/357 (12%) 49/348 (14%)	9.0/11.3	NR	NR	NR	25.2/6.2	55.0/10.4	NR	19.9/7.0
LMCA + SVD, %	67/357 (19%) 71/348 (20%)	16.7/17.7	NR	NR	13.0/6.0	24.0/10.5	21.0/14.4	NR	27.3/15.9
LMCA + DVD, %	112/357 (31%) 106/348 (30%)	33.7/30.0	NR	NR	27.0/19.0	26.0/26.3	16.0/22.4	NR	30.3/25.9
LMCA + TVD, %	136/357 (38%) 122/348 (35%)	40.7/41	NR	NR	60.0/75.0	24.8/57.0	8.0/52.8	NR	22.5/51.2
2-Vessel disease, %	NR	NR	60.0./58.0	42.0/42.0	NR	NR	NR	NR	NR
3-Vessel disease, %	60.0/61.0	NR	40.0/42.0	58.0/58.0	NR	NR	NR	NR	NR
Type of stent	PES	SES	BMS	BMS	BMS/DES	BMS/DES	BMS	NR	DES

Data are presented as percentage treated with PCI/percentage treated with CABG, unless otherwise stated

*ASAN-MAIN* ASAN Medical Center-Left MAIN Revascularization; *BMI* Body mass index; *BMS* Bare metal stent; *CVA* Cerebrovascular disease; *CABG* Coronary artery bypass graft; *DVD* Double-vessel disease; *DES* Drug-eluting stent; *LMCA* Left main coronary artery; *LVEF* Left ventricular ejection fraction; *LE MANS* Left Main Stenting Trail; *MASS-II* Medicine, Angioplasty, or Surgery Study; *MAIN-COMPARE* Revascularization for Unprotected Left Main Coronary ArteryStenosis: Comparison of Percutaneous Coronary Angioplasty versus Surgical Revascularization; *MI* Myocardial infarction; *NR* No record; *PCI* Percutaneous coronary intervention; *PES* Paclitaxel-eluting stent; *PRECOMBAT* Premier of Randomized Comparison of Bypass Surgery;. Angioplasty Using Sirolimus-Eluting Stent in Patients with Left Main Coronary Artery Disease; *RCT* Randomizedcontrolled trial; *SVD* Single-vessel disease; *SES* Sirolimus-eluting stent; *SOS* Stent or Surgery; *SYNTAX* Synergy Between Percutaneous Coronary Intervention with TAXUS and Cardiac Surgery; *TVD* Triple-vessel disease

### All-cause mortality

**Fig. 2:**
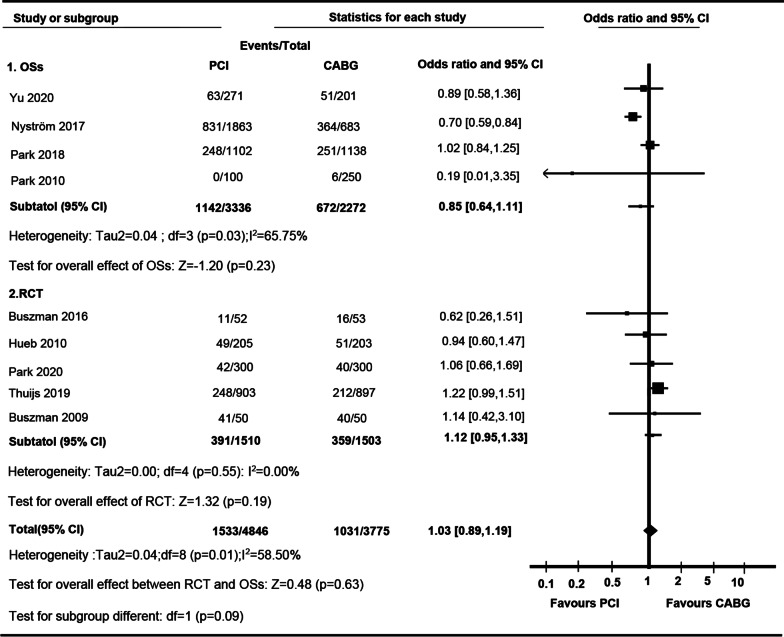
Forest plot of OR of the composite of All-cause Mortality at follow-up for PCI vs. CABG (*ASAN-MAIN* ASAN Medical Center-Left MAIN Revascularization; *CABG* Coronary artery bypass graft; *LE MANS* Left main stenting trail; *MASS-II* Medicine, Angioplasty, or Surgery Study; *MAIN-COMPARE* Revascularization for unprotected left main coronary artery stenosis: Comparison of Percutaneous Coronary Angioplasty versus Surgical Revascularization; *OSs* Observational study; *PCI* Percutaneous coronary intervention; *PRECOMBAT* Premier of randomized comparison of bypass surgery; Angioplasty using sirolimus-eluting stent in patients with left main coronary artery disease; *RCT* Randomized controlled trial; *SOS* Stent or surgery; *SYNTAX* Synergy between percutaneous coronary intervention with TAXUS and cardiac surgery)

There was no significant difference between PCI and CABG in the incidence of all-cause mortality in RCTs {Odds Ratio (OR) 1.12 [95% confidence interval (CI) 0.95 to 1.33]; I^2^ = 0.00%} or cohort studies (OR 0.85 [95% CI 0.64 to 1.11]; I^2^ = 65.75%) or in total (OR 1.03 [95% CI 0.89**–**1.19]; I^2^ = 58.5%) (Fig. 2). And a statistical significance of OR was not observed between RCTs and OSs (P for interaction, 0.09). Due to the existence of heterogeneity, subgroup and sensitivity tests were performed in the categorization based on lesion location of patient’s baseline (LMCA or MCA) or types of stents used in the studies (DES or BMS). Significant difference between PCI and CABG in terms of LMCA (OR 0.93 [95% CI 0.72 to 1.19]) and MVCA (OR 0.99 [95% CI 0.83 to 1.19]), DES (OR 1.05 [95% CI 0.85 to 1.30]) and BMS (OR 0.83 [95% CI 0.24 to 2.83]) was not observed (Additional file 1: Tables S3 and S4).

### Cardiac mortality

**Fig. 3:**
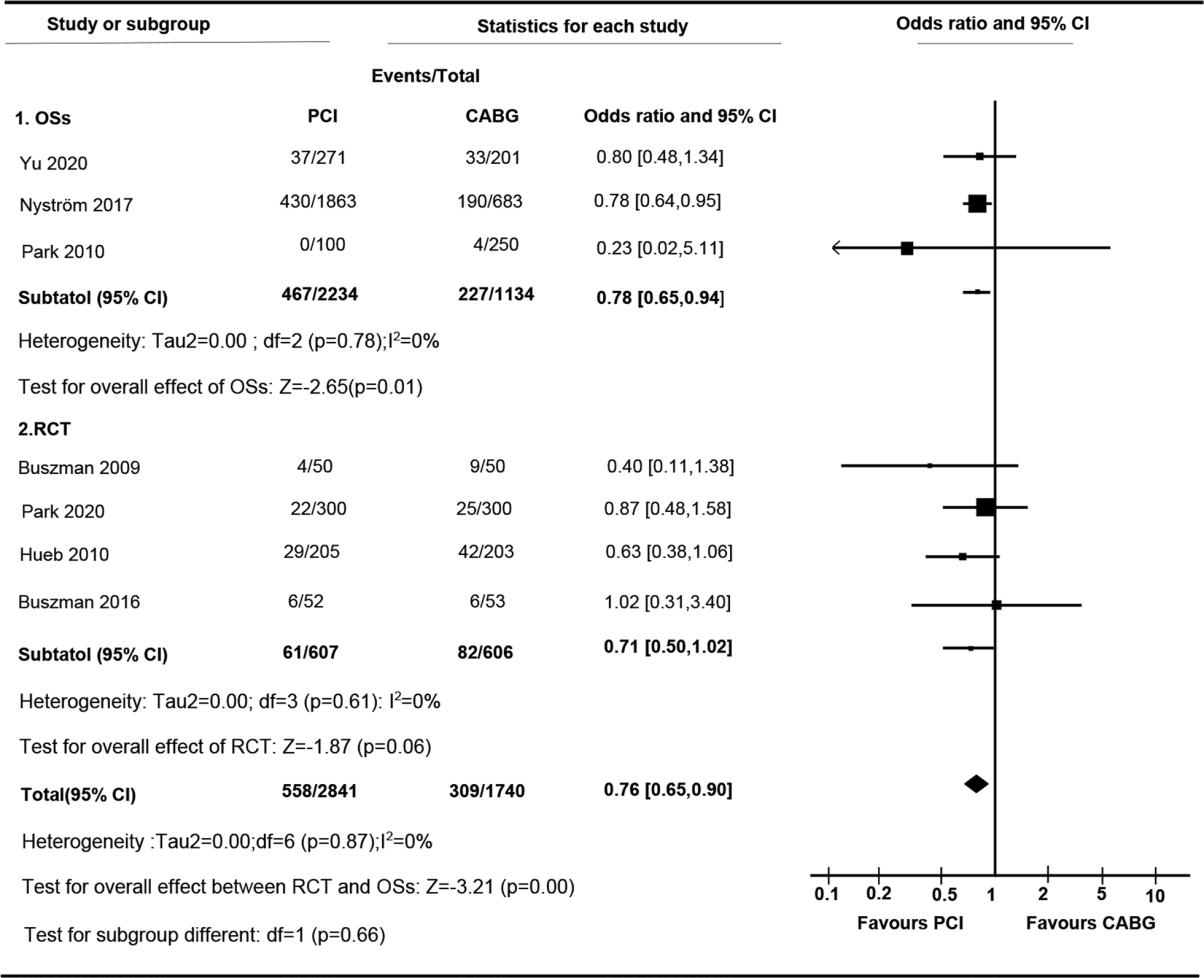
Forest plot of OR of the composite of Cardiac Mortality at follow-up for PCI vs. CABG (*ASAN-MAIN* ASAN Medical Center-Left MAIN Revascularization; *CABG* Coronary artery bypass graft; *LE MANS* Left main stenting trail; *MASS-II* Medicine, Angioplasty, or Surgery Study; *OSs* Observational study; *PCI* Percutaneous coronary intervention; *PRECOMBAT* Premier of randomized comparison of bypass surgery; Angioplasty Using Sirolimus-Eluting Stent in Patients with Left Main Coronary Artery Disease; *RCT* Randomized controlled trial; *SOS* Stent or surgery)

There was a significant increase of cardiac morality rate in PCI comparing to CABG (OR 0.76 [95% CI 0.65 to 0.90]; I^2^ = 0.00%) (Fig. 3). Difference of cardiac morality between PCI and CABG in cardiac mortality in RCTs (OR 0.71 [95% CI 0.50 to 1.02]; I^2^ = 0.00%) was not observed, whereas PCI was associated with higher incidence in OSs (OR 0.78 [95% CI 0.65 to 0.94]; I^2^ = 0.00%). There was no significant difference in OR between RCTs and OSs (P for interaction, 0.66). Although statistic heterogeneity was not observed, subgroup studies were still performed to analyze potential clinical differences within studies. The results showed that there was no significant difference in cardiac mortality between PCI and CABG for LMCA (OR 0.90.95% CI 0.53 to 1.53). However, the difference in cardiac mortality between PCI and CABG for MVCA was statistically significant (OR 0.59 [95% CI 0.37 to 0.95]) (Additional file 1: Table S3).

### Repeated revascularization

**Fig. 4:**
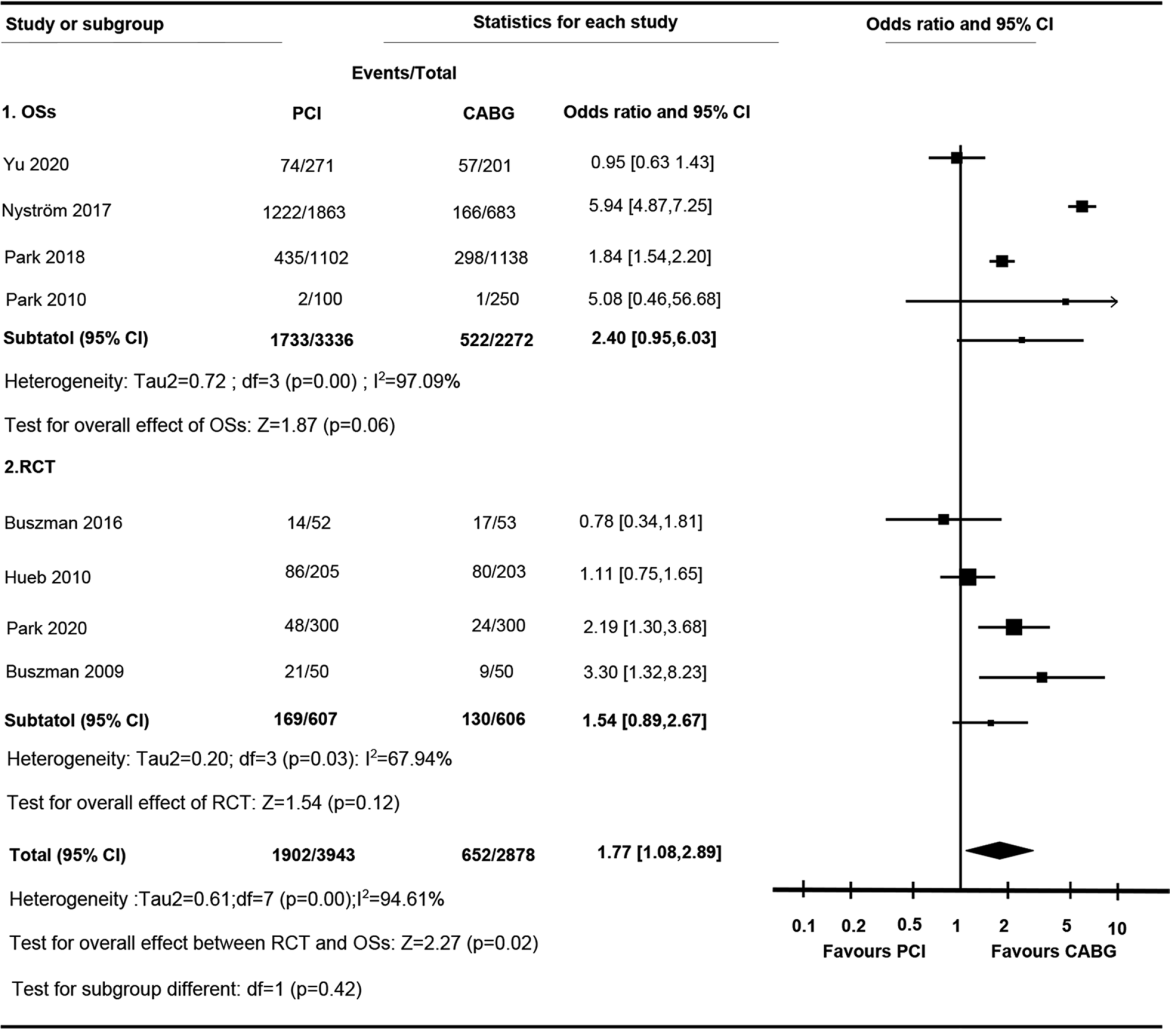
Forest plot of OR of the composite of Repeated Revascularization at follow-up for PCI vs. CABG (*ASAN-MAIN* ASAN Medical Center-Left MAIN Revascularization; *CABG* Coronary artery bypass graft; *LE MANS* Left main stenting trail; *MASS-II* Medicine, Angioplasty, or Surgery Study; *MAIN-COMPARE* Revascularization for unprotected left main coronary artery stenosis: Comparison of Percutaneous Coronary Angioplasty versus Surgical Revascularization; *OSs* Observational study; *PCI* Percutaneous coronary intervention; *PRECOMBAT* Premier of randomized comparison of bypass surgery; Angioplasty Using Sirolimus-Eluting Stent in Patients with Left Main Coronary Artery Disease; *RCT* Randomized controlled trial; *SOS* Stent or surgery)

PCI was associated with higher rate of repeated revascularization rate comparing to CABG (OR 1.77 [95% CI 1.08 to 2.89]; I^2^ = 94.61%) (Fig. 4). The rate of repeat revascularization between PCI and CABG was not significantly different in RCTs (OR 1.54 [95% CI 0.89 to 2.67] ; I^2^ = 67.9%) and OSs (OR 2.40 [95% CI 0.95 to 6.03] ; I^2^ = 97.1%). There was no significant difference in OR between RCTs and OSs (P for interaction, 0.42). Subgroup tests were performed owing to heterogeneity, showing that no substantial difference between LMCA (OR 1.38 [95%, 0.51 to 3.78]) and MVCA (OR 1.77 [95%, 0.62 to 5.07]) was detected (Additional file 1: Tables S3 and S4). In contrast to DES (OR 1.40 [95% CI 0.68 to 2.87]), BMS was associated with higher incidence of repeated revascularization than CABG in OSs (OR 4.16 [95% CI 3.07 to 5.64]).

### Stroke

**Fig. 5:**
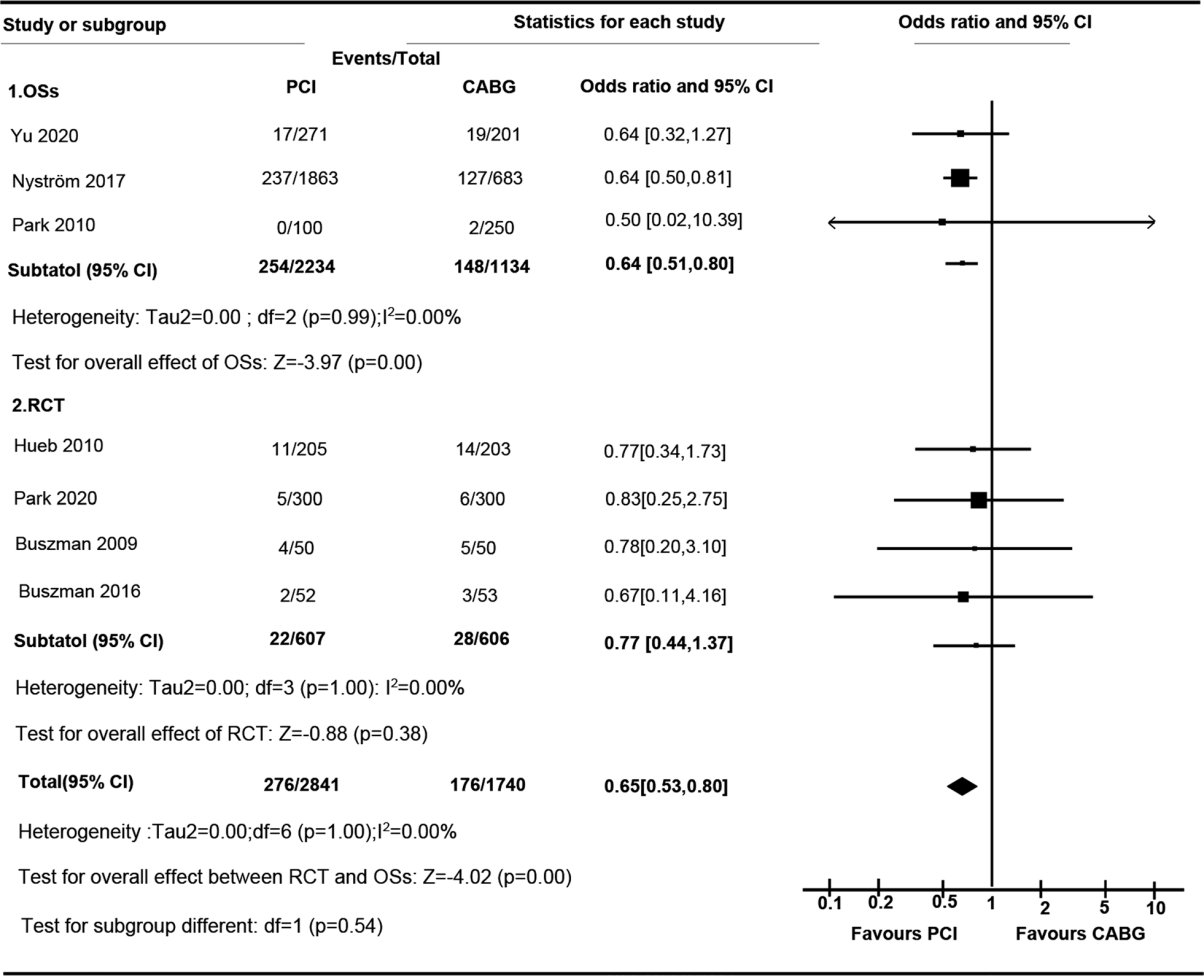
Forest plot of OR of the composite of Stroke at follow-up for PCI vs. CABG (*ASAN-MAIN* ASAN Medical Center-Left MAIN Revascularization; *CABG* Coronary artery bypass graft; *LE MANS* Left main stenting trail; *MASS-II* Medicine, Angioplasty, or Surgery Study; *OSs* Observational study; *PCI* Percutaneous coronary intervention; *PRECOMBAT* Premier of Randomized Comparison of Bypass Surgery; Angioplasty Using Sirolimus-Eluting Stent in Patients with Left Main Coronary Artery Disease; *RCT* Randomized controlled trial; *SOS* Stent or surgery)

There was a significant increase of stroke rate in CABG comparing to PCI (OR 0.65 [95% CI 0.53 to 0.80]; I^2^ = 0.00%) (Fig. 5). In cohort studies, incidence of stroke in PCI group was lower than those in CABG (OR 0.64 [95% CI 0.51 to 0.80]; I^2^ = 0.00%), whereas no significant difference between PCI and CABG in RCT was observed (OR 0.77 [95% CI 0.44 to 1.37]; I = 0.00%). Difference of OR was not observed between RCTs and OSs (P for interaction, 0.54). In subgroup studies, there was not significant difference between LMCA (OR 0.78 [95% CI 0.29 to 2.12]) and MVCA (OR 0.77 [95% CI, 0.38 to 1.55]) (P = 1.00) (Additional file 1: Table S3) (Fig. 5).

### Myocardial infarction

**Fig. 6:**
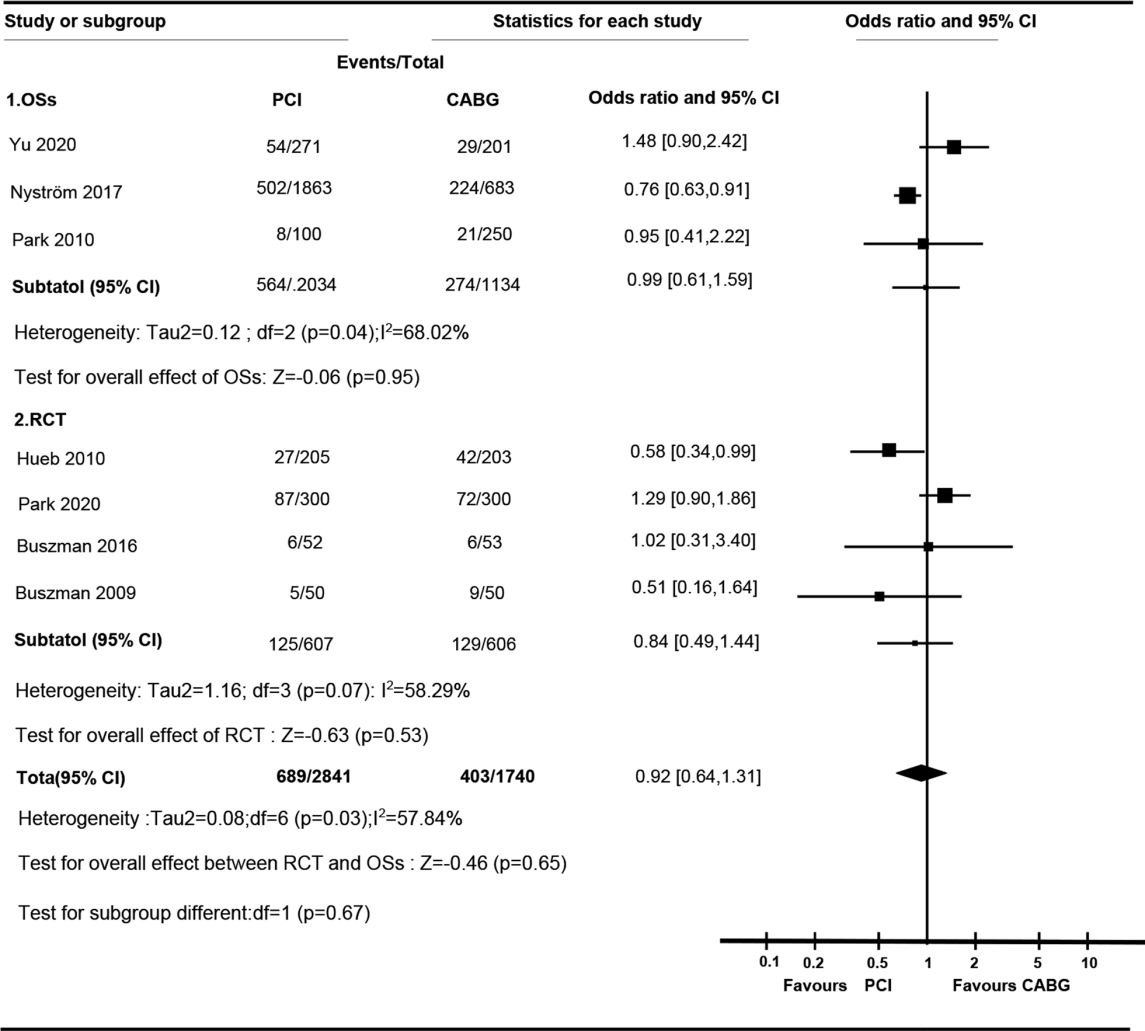
Forest plot of OR of the composite of Myocardial Infarction at follow-up for PCI vs. CABG (*ASAN-MAIN* ASAN Medical Center-Left MAIN Revascularization; *CABG* Coronary artery bypass graft; *LE MANS* Left main stenting trail; *MASS-II* Medicine, Angioplasty, or Surgery Study; *OSs* Observational study; *PCI* Percutaneous coronary intervention; *PRECOMBAT* Premier of randomized comparison of bypass surgery; Angioplasty Using Sirolimus-Eluting Stent in Patients with Left Main Coronary Artery Disease; *RCT* Randomized controlled trial; *SOS* Stent or surgery)

No significant difference between PCI and CABG in myocardial infarction in RCTs (OR 0.84 [95% CI, 0.49 to 1.44]; I^2^ = 58.30%) or cohort studies (OR, 0.99 [95% CI 0.61 to 1.59]; I^2^ = 68.02%), or in total (OR 0.92 [95% CI 0.64 to 1.31]; I^2^ = 57.84%) was not observed. Statistical difference of OR was not observed between RCTs and cohort studies (P for interaction, 0.67). Due to the existence of heterogeneity, subgroup tests were performed. Comparing to PCI, CABG was associated with higher rate of myocardial infarction in MVCA (OR 0.57 [95% CI 0.25 to 0.92]), while there was no significant difference between PCI and CABG in LMCA (OR 0.78 [95%, 0.29 to 2.12]). Significant difference was observed between LMCA and MVCA (P = 0.01) (Additional file 1: Table S4) (Fig. 6).

## Discussion

To our best knowledge, our study is the first meta-analysis to present 10-year comparative outcomes between PCI and CABG for treating patients with either LMCA or MVCA. We tried to overcome potential problems that might weaken the credibility of clinical evidence in previous studies. For example, in a meta-analysis of left main coronary artery, data from OSs and RCTs was pooled together without classification, leading to a less credible conclusion due to the internal difference between 2 types of studies [22]. For the same reason, unlike obscure definition of CAD in a meta-analysis involving 23 RCTs, we primarily focused on CAD with LMCA or MVCA, which is more clinical applicable [23].

In our study, after 10-year follow-up, there was no significant difference of all-cause mortality rate between PCI and CABG in treating MVCA or LMCA in both RCTs and OSs group, as well as in subgroups categorized by lesion locations or types of PCI. In our subgroup analysis of mortality, PCI was not associated with better outcome in either LMCA or MVCA subgroups. Previous publications showed that CABG had a mortality benefit over PCI among patients with MVCA, but not among patients with LMCA in both short-term and long-term follow-up studies [5, 24]. Combining the results from our study and prior studies, it showed that CABG improved 5-year survival but not 10-year survival among patients with MVCA. In the contrast, there was no significant difference between CABG and PCI for treating patients with LMCA in either 5-year or 10-year survival rate.

Different results between our studies and previous studies in terms of all-cause death rate could be explained by the discrepancy of patients’ lesion complexity in the 2 groups. For example, an included study showed that CABG group has higher rates of myocardial infarction history, left main plus triple-vessel disease and chronic total occlusion than PCI group [20]. The same situation appeared in another 2 included studies, as patients undergoing CABG were with higher clinical and anatomic risk factor profiles than those treated by PCI [18, 19]. Thus, the therapeutic effect of CABG might be underpowered due to the fact that the lesion of CAD among patients treated by CABG was often associated with higher-risk clinical and angiographic situation than that among those treated by PCI [15, 21]. Except Synergy Between Percutaneous Coronary Intervention with TAXUS and Cardiac Surgery (SYNTAX) trail and Left Main Stenting (LE MANS) trail, other included studies were lack of syntax score evaluation, especially in the observational studies, leading to a unquantifiable and unstandardized process for patients’ selection.

In addition, comparing to CABG, our results suggested that PCI was associated with higher incidence in cardiac mortality, but with lower incidence of cardiac death among patients with MVCA. These findings contradicted the common perception based on the evidence from 5-year data of SYNTAX trail [25]. Following reasons may play roles in the explanation of this contradiction. Firstly, after investigation for underlying reasons, we identified an included OS which only enrolled diabetic patients might affect the effect size of final result [21]. Except this study, only 20 ~ 30% candidates in the PCI and CABG group from other 2 included studies had diabetes. We did sensitivity test by excluding the study that primarily focused on diabetic patients [26]. After the exclusion of that study, we found that there was no difference between PCI and CABG in cardiac mortality, which was in line with previous findings. Secondly, the application of dual antiplatelet therapy after PCI was found to reduce the incidence of death and myocardial infarction in some measure, causing the equivalent cardiac death rate between PCI and CABG [27].

Our results in terms of lower incidence of stroke in PCI group at 10-year follow-up was consistent with previous findings from an individual patient data meta-analysis of 11 RCTs at 3.3-year follow-up, showing that CABG had a significantly higher stroke incidence at 5-year follow-up [28]. Multifactor were likely to contribute to the increased risk of stroke with CABG. The usage of on-pump and off-pump in surgical procedures, intra-operative hypoperfusion, strategies for post-operative bleeding control and post-operative atrial fibrillation might lead to higher stroke risk with surgery. However, in the latest revealed RCT comparing PCI to CABG, there were no obvious differences of stroke rate between 2 groups, as the usage of postoperative double-antiplatelet rates reached up to 45% which was higher or at least consistent with data from previous large RCTS [6]. In our subgroup study, we also found that BMS was associated with higher incidence of repeated revascularization than DES, which is in line with findings from previous publications [29, 30].

### Limitation

Few limitations including the synthesis of heterogeneous trials in terms of variable study design and characteristics were detected. For this reason, we utilized random-effects model and heterogeneity test to reduce statistics bias. What’s more, there was one included RCTwith9.6 ± 0.85 years follow-up on average [14], and another included OS with candidates who were all diabetic and MVCA patients [21]. We performed sensitivity and subgroup analyses showing that all results remained robust when excluding these 2 studies. Besides, thanks to limited numbers of studies and data in this area, we were unable to perform meta-regression to find clinical predictors for better outcomes after treatment. SYNTAX score has been considered as an important predictor for higher survival rate as both 5-year individual data from 11 RCTs and 10-year data from SYNTAX trail confirmed that CAD patients with SYNTAX score ≥ 33 treated by CABG had lower mortality rate than PCI [5, 24]. However, only 2 included trails had SYNTAX score so that we were unable to further explore the impact of SYNTAX score on the patients’ outcome after treated by PCI or CABG.

## Conclusion

Evidence from our study and prior studies suggested the superiority of CABG over PCI in improving 5- but not 10-year survival among patients with MVCA. In the contrast, there was no significant difference between CABG and PCI for treating patients with LMCA in either 5- or 10- years survival rate.

## Supplementary Information

**Additional file 1.** Supplemental material.

## Data Availability

The data that support the findings of this study are available from the corresponding author upon reasonable request.

## Abbreviations

BMS: Bare metal stent
CABG: Coronary artery bypass grafting
CAD: Coronary artery disease
CI: Confidence interval
DES: Drug-eluting stent
LMCA: Left main coronary artery
MACCE: Major adverse cardiac and cerebrovascular event
MVCA: Multivessel coronary artery
OSs: Observational studies
RCT: Randomized controlled trial
