# Supplementary material for: Ten-year outcomes after percutaneous coronary intervention versus coronary artery bypass grafting for multivessel or left main coronary artery disease: a systematic review and meta-analysis
Source: J Cardiothorac Surg. 2023 Feb 2;18:54. doi: 10.1186/s13019-023-02101-y (PMC9893531; doi:10.1186/s13019-023-02101-y)
Supplement: Supplementary file 1 — Additional file 1. Supplemental material. [file 13019_2023_2101_MOESM1_ESM.doc]

Supplemental material

**Context**

1. Searching Algorithm………………………………………...3
2. Inclusion and Exclusion Criteria……………………………5
3. Primary and Major Secondary Endpoint Definitions……….6
4. Included Studies and Baseline Information…………………8
5. Quality Analysis and Bias Assessment…………………….10
6. Subgroup and Sensitivity Analysis…………………………12

**A. Searching Algorithm**

For **PUBMED**, the search used was:

((((((((((((((((((((((((((((((((((coronary heart disease) OR (Coronary Diseases)) OR (Disease, Coronary)) OR (Coronary Heart Disease)) OR (Coronary Heart Diseases)) OR (Disease, Coronary Heart)) OR (Diseases, Coronary Heart)) OR (Heart Disease, Coronary)) OR (Heart Diseases, Coronary)) AND (Percutaneous Coronary Intervention)) OR (Coronary Intervention, Percutaneous)) OR (Coronary Interventions, Percutaneous)) OR (Intervention, Percutaneous Coronary)) OR (Interventions, Percutaneous Coronary)) OR (Percutaneous Coronary Interventions)) OR (Percutaneous Coronary Revascularization)) OR (Coronary Revascularization, Percutaneous)) OR (Coronary Revascularizations, Percutaneous)) OR (Percutaneous Coronary Revascularizations)) OR (Revascularization, Percutaneous Coronary)) OR (Revascularizations, Percutaneous Coronary)) OR (PCI)) AND (Coronary Artery Bypass Graft)) OR (Artery Bypass, Coronary)) OR (Artery Bypasses, Coronary)) OR (Bypasses, Coronary Artery)) OR (Coronary Artery Bypasses)) OR (Coronary Artery Bypass Surgery)) OR (Bypass, Coronary Artery)) OR (Aortocoronary Bypass)) OR (Aortocoronary Bypasses)) OR (Bypass, Aortocoronary)) OR (Bypasses, Aortocoronary)) OR (Bypass Surgery, Coronary Artery)) OR (Coronary Artery Bypass Grafting) AND (“last 20 years"[PDat])

For **EMBASE**, the search used was:

#1 ‘ischemic heart disease’/exp OR ‘coronary heart disease’ OR ‘Coronary Diseases’ OR ‘Disease, Coronary’ OR ‘Coronary Heart Disease’ OR ‘Coronary Heart Diseases’ OR ‘Disease, Coronary Heart’ OR ‘Diseases, Coronary Heart’ OR ‘Heart Disease, Coronary’ OR ‘Heart Diseases, Coronary’:ab,ti

#2 ‘Percutaneous Coronary Intervention’ /exp OR ‘Coronary Intervention, Percutaneous’ OR ‘Coronary Interventions, Percutaneous’ OR ‘Intervention, Percutaneous Coronary’ OR ‘Interventions, Percutaneous Coronary’ OR ‘Percutaneous Coronary Interventions’ OR ‘Percutaneous Coronary Revascularization’ OR ‘Coronary Revascularization, Percutaneous’ OR ‘Coronary Revascularizations, Percutaneous’ OR ‘Percutaneous Coronary Revascularizations’ OR ‘Revascularization, Percutaneous Coronary’ OR ‘Revascularizations, Percutaneous Coronary’ OR ‘PCI’ :ab,ti

#3’Coronary Artery Bypass Graft’/exp OR ‘Artery Bypass, Coronary’ OR ‘Artery Bypasses, Coronary’ OR ‘Bypasses, Coronary Artery’ OR ‘Coronary Artery Bypasses’ OR ‘Coronary Artery Bypass Surgery’ OR ‘Bypass, Coronary Artery’ OR ‘Aortocoronary Bypass’ OR ‘Aortocoronary Bypasses’ OR ‘Bypass, Aortocoronary’ OR ‘Bypasses, Aortocoronary’ OR ‘Bypass Surgery, Coronary Artery’ OR ‘Coronary Artery Bypass Grafting’ :ab,ti

#4 #1 AND #2 AND #3 AND [2000-2021]/py

For **COCHRANE**, the search used was:

#1 MeSH descriptor：[Coronary Disease] explode all trees

#2 (Coronary Disease):ti,ab,kw OR (Disease,Coronary) :ti,ab,kw OR (Coronary Heart Disease) :ti,ab,kw OR (Coronary Heart Diseases) :ti,ab,kw OR (Disease,Coronary Heart) :ti,ab,kw OR (Diseases,Coronary Heart) :ti,ab,kw OR (Heart Disease,Coronary) :ti,ab,kw OR (Heart Diseases,Coronary) :ti,ab,kw

#3 #1 OR #2

#4 MeSHdescriptor: [Percutaneous Coronary Intervention] explode all trees

#5 (Coronary Intervention ,Percutaneous) :ti,ab,kw OR (Coronary Interventions ,Percutaneous) :ti,ab,kw OR (Intervention ,Percutaneous Coronary) :ti,ab,kw OR (Interventions ,Percutaneous Coronary) :ti,ab,kw OR (Percutaneous Coronary Interventions) :ti,ab,kw OR (Percutaneous Coronary Revascularization) :ti,ab,kw OR (Coronary Revascularization ,Percutaneous) :ti,ab,kw OR (Coronary Revascularizations ,Percutaneous) :ti,ab,kw OR (Percutaneous Coronary Revascularizations) :ti,ab,kw OR (Revascularization,Percutaneous Coronary) :ti,ab,kw OR (Revascularizations,Percutaneous Coronary) :ti,ab,kw

#6 #4 OR #5

#7 MeSH descriptor: (Coronary Artery Bypass) explode all trees

#8 (Artery Bypass ,Coronary) :ti,ab,kw OR (Artery Bypasses ,Coronary) :ti,ab,kw OR (Bypass ,Coronary Artery) :ti,ab,kw OR (Bypasses ,Coronary Artery) :ti,ab,kw OR (Coronary Artery Bypasses) :ti,ab,kw OR (Coronary Artery Bypass Surgery) :ti,ab,kw OR (Bypass Surgery ,Coronary Artery) :ti,ab,kw OR (Aortoconronary Bypasses) :ti,ab,kw OR (Bypass Aortoconronary) :ti,ab,kw OR (Bypasses Aortoconronary) :ti,ab,kw OR (Aortoconronary Bypass) :ti,ab,kw OR (Coronary Artery Bypass Grafting) :ti,ab,kw OR (Coronary Artery Bypass Graft) :ti,ab,kw

#9 #7 OR #8

#10 #3 AND #6 AND #9

**B. Inclusion and Exclusion Criteria**

1. Inclusion:

1) PCI or CABG in treating artery stenosis of either unprotected left main coronary artery or multi-vessel coronary artery with more than 10 years follow-up

2) the study design may be any of the following: randomized controlled trials, prospective and retrospective cohort studies

2. Exclusion:

1) with prior cardiothoracic surgery

2) acute myocardial infarction

3) significant valvular heart disease

4) less than 10 participants in either group

5) studies without primary outcomes (all-cause death)

**C. Primary and Major Secondary Endpoint Definitions**

**Death**：

The primary end point includes death from any cause. In addition, the cause of death (cardiac vs. non-cardiac) will be adjudicated. If the cause of death cannot be adjudicated the most severe cause will be considered.

**Cardiac death** is defined as death due to any of the following:

1. Acute myocardial infarction.

2. Cardiac perforation/pericardial tamponade.

3. Arrhythmia or conduction abnormality.

4. Cerebrovascular accident within 30 days of the procedure or cerebrovascular accident suspected of being related to the procedure.

5. Death due to complication of the procedure

6. Any death in which a cardiac cause cannot be excluded.

**Non-cardiacdeath** is defined as a death not due to cardiac causes (as defined above), including bleeding-related death.

**Myocardial Infarction：**

MI was defined as the appearance of both new Q-waves and an increase in the creatine kinase MB level to greater than five times the upper limit of the normal range, if occurring within 48 hours after the procedure, or as the appearance of either new Q-waves or an increase in the creatine kinase MB level to greater than the upper limit of the normal range, plus ischemic symptoms or signs, if occurring more than 48 hours after the procedure.

**Stroke ：**

Stroke was defined as a sudden onset of neurologic deficit (i.e., vertigo, numbness, aphasia, or dysarthria) resulting from vascular lesions of the brain, including hemorrhage, embolism, thrombosis, or rupturing aneurysm, and persisting for >24 hours.

**Ischemia-driven revascularization：**

Target-vessel revascularization, in which repeat revascularization with either percutaneous coronary intervention (PCI) or coronary artery bypass grafting (CABG) was performed in the treated vessel, was considered to be driven by ischemia if the stenosis of any vessel was at least 50% of the diameter of the vessel in the presence of ischemic signs or symptoms or if the stenosis was at least 70% of the diameter of the vessel regardless of the presence or absence of ischemic signs or symptoms.Any coronary revascularization procedure may be either a PCI or a CABG.

**PCI:**

Percutaneous coronary intervention is a procedure used to treat narrowing of the coronary arteries of the heart found in coronary artery disease. The process involves combining coronary angioplasty with stenting, which is the insertion of a permanent wire meshed tube that is either eluting or composed of bare metal.

**CABG:**

A surgical procedure performed to shunt blood around a narrowing or blockage in the [coronary artery](https://www.merriam-webster.com/dictionary/coronary artery) of the heart that usually involves grafting one end of a segment of blood vessel (such as a vein of the leg) removed from another part of the body into the aorta and the other end of the segment into the coronary artery beyond the obstructed area to allow for increased blood flow.

**Multivessel Coronary Artery Disease:**

MVDdefined as a diameter stenosis of more than 70% in 2 or more major epicardial vessels involving at least 2 separate coronary artery territories and without left main coronary artery disease.

**Left Main Coronary Artery**：

De novo LMCA stenosis≥50%or left main equivalent disease defined as ≥50% stenosis of the ostium of the left anterior descending and left circumflex with or without stenosis in other vessels.

**D. Quality Analysis**

|  | MASS II | PRECOMBAT | SYNTAX | SOS | LE MANS |
| --- | --- | --- | --- | --- | --- |
| Random sequence generation (selection bias) | ？ | + | + | + | ？ |
| allocation concealment (selection bias) | ？ | + | + | + | ？ |
| blinding of participants and personnel (performance bias) | - | - | - | - | - |
| Incomplete outcome data (attrition bias) | + | + | - | - | + |
| Selective reporting (reporting bias) | + | + | + | + | + |
| other potential bias | + | + | + | + | + |
| **Table 1 Assessment of the risks of different bias for RCTs by using Cochrane risk of bias tool.**  LE MANS: Left Main Stenting Trial; MASS-II: Medicine, Angioplasty, or Surgery Study; PRECOMBAT: Premier of Randomized Comparison of Bypass Surgery vsAngioplasty Using Sirolimus-Eluting Stent in Patients With Left Main CoronaryArtery Disease; SOS: Stent or Surgery; SYNTAX: Synergy Between PCI With Taxus and CardiacSurgery. | | | | | |

| Study | selection |  |  |  | Comparability | outcome |  |  | Score |
| --- | --- | --- | --- | --- | --- | --- | --- | --- | --- |
|  | Representativeness of exposed cohort | Selection of the non-exposed cohort | Ascertainment of exposure | Demonstration that outcome of interest was not present at start of study |  | Assessment of outcome | Was follow-up long enough for outcomes to occur | Adequacy of follow-up of cohorts |  |
| Yu 2020 | 1 | 1 | 1 | 0 | 1 | 1 | 1 | 1 | 7 |
| Nyström 2017 | 1 | 1 | 1 | 0 | 1 | 1 | 1 | 1 | 7 |
| MAIN-COMPARE | 1 | 1 | 1 | 0 | 0 | 1 | 1 | 1 | 6 |
| ASAN-MAIN | 1 | 1 | 1 | 0 | 0 | 1 | 1 | 1 | 6 |
| **Table 2 Assessment of the risks of different bias for RCTs by using Newcastle-Ottawa Scale.**  ASAN-MAIN：ASAN Medical Center–Left MAIN Revascularization；MAIN-COMPARE: Revascularization for Unprotected Left Main Coronary ArteryStenosis: Comparison of Percutaneous Coronary Angioplasty versus Surgical Revascularization. | | | | | | | | | |

1. **Subgroup and Sensitivity Analysis**

|  |  |  |  |  | Random-effects model | | | Heterogeneity | |
| --- | --- | --- | --- | --- | --- | --- | --- | --- | --- |
|  | Outcomes | Study type | No. of studies | No. of patients (PCI/CABG) | OR (95% CI) | P value | P value for interaction | I^2^, % | P value |
| All cause morality | LMCA | RCT | 3 | 709/701 | 0.93 (0.72, 1.19) | 0.54 | 0.19 | 0.00 | 0.58 |
|  |  | OS | 3 | 1473/1589 | 0.99 (0.83, 1.19) | 0.95 | NA | 0.00 | 0.44 |
|  | MCA | RCT | 3 | 801/802 | 1.26(0.86, 1.84) | 0.24 | 0.19 | 88.05 | 0.14 |
|  |  | OS | 1 | 1863/683 | NA | NA | NA | NA | NA |
| Repeated revascularization | LMCA | RCT | 2 | 352/353 | 1.38 (0.51, 3.78) | 0.53 | 0.76 | 76.13 | 0.57 |
|  |  | OS | 3 | 1473/1589 | 1.47 (0.80, 2.70) | 0.22 | NA | 78.57 | 0.09 |
|  | MCA | RCT | 2 | 255/253 | 1.77 (0.62, 5.07) | 0.29 | 0.76 | 78.20 | 0.03 |
|  |  | OS | 1 | 1863/683 | NA | NA | NA | NA | NA |
| Cardiac morality | LMCA | RCT | 2 | 352/353 | 0.90 (0.53, 1.53) | 0.70 | 0.43 | 0.00 | 0.82 |
|  |  | OS | 2 | 371/451 | 0.78 (0.47, 1.29) | 0.33 | NA | 0.00 | 0.48 |
|  | MCA | RCT | 2 | 255/253 | 0.59 (0.37, 0.95) | 0.03 | 0.43 | 0.00 | 0.50 |
|  |  | OS | 1 | 1863/683 | NA | NA | NA | NA | NA |
| Stroke | LMCA | RCT | 2 | 352/353 | 0.78 (0.29, 2.12) | 0.62 | 1.00 | 0.00 | 0.84 |
|  |  | OS | 2 | 371/451 | 0.63 (0.33, 1.23) | 0.18 | NA | 0.00 | 0.87 |
|  | MCA | RCT | 2 | 255/253 | 0.77 (0.38, 1.55) | 0.47 | 1.00 | 0.98 | NA |
|  |  | OS | 1 | 1863/683 | NA | NA | NA | NA | NA |
| Myocardial infarction | LMCA | RCT | 2 | 352/353 | 0.78 (0.29, 2.12) | 0.62 | 0.01 | 0.00 | 0.84 |
|  |  | OS | 2 | 371/451 | 0.63 (0.33, 1.23) | 0.18 | NA | 0.00 | 0.87 |
|  | MCA | RCT | 2 | 255/253 | 0.57 (0.25, 0.92) | 0.02 | 0.01 | 0.00 | NA |
|  |  | OS | 1 | 1863/683 | NA | NA | NA | NA | NA |
| **Table 3 Subgroup and sensitivity test（lesion location）.**  RCT: randomized clinical trial; OS: observational study; LMCA: Left main coronary artery; MCA: Multivessel coronary artery disease; NA: not applicable;  *P for interaction: difference between different subgroup in the same study design. | | | | | | | | | |

|  |  |  |  |  | Random-effects model | | | Heterogeneity | |
| --- | --- | --- | --- | --- | --- | --- | --- | --- | --- |
|  | Outcomes | Study type | No. of studies | No. of patients (PCI/CABG) | OR (95% CI) | P value | P value for interaction | I^2^, % | P value |
| All cause morality | DES | RCT | 2 | 1203/1197 | 1.19 (0.98, 1.45) | 0.07 | NA | 0.00 | 0.58 |
|  |  | OS | 2 | 1055/891 | 1.05 (0.85, 1.30) | 0.67 | 0.37 | 0.00 | 0.39 |
|  | BMS | RCT | 0 | NA | NA | NA | NA | NA | NA |
|  |  | OS | 2 | 418/698 | 0.83 (0.24, 2.83) | 0.76 | 0.37 | 28.30 | 0.24 |
| Repeated revascularization | DES | RCT | 1 | 300/300 | NA | NA | NA | NA | NA |
|  |  | OS | 2 | 1055/891 | 1.40 (0.68, 2.87) | 0.36 | 0.01 | 89.62 | 0.00 |
|  | BMS | RCT | 0 | NA | NA | NA | NA | NA | NA |
|  |  | OS | 2 | 418/698 | 4.16 (3.07, 5.64) | 0.00 | 0.01 | 0.00 | 0.87 |
| Cardiac morality | DES | RCT | 1 | 300/300 | NA | NA | NA | NA | NA |
|  |  | OS | 1 | 271/201 | NA | NA | NA | NA | NA |
|  | BMS | RCT | 0 | NA | NA | NA | NA | NA | NA |
|  |  | OS | 1 | 100/250 | NA | NA | NA | NA | NA |
| Stroke | DES | RCT | 1 | 300/300 | NA | NA | NA | NA | NA |
|  |  | OS | 1 | 271/201 | NA | NA | NA | NA | NA |
|  | BMS | RCT | 0 | NA | NA | NA | NA | NA | NA |
|  |  | OS | 1 | 100/250 | NA | NA | NA | NA | NA |
| Myocardial infarction | DES | RCT | 1 | 300/300 | NA | NA | NA | NA | NA |
|  |  | OS | 1 | 271/201 | NA | NA | NA | NA | NA |
|  | BMS | RCT | 0 | NA | NA | NA | NA | NA | NA |
|  |  | OS | 1 | 100/250 | NA | NA | NA | NA | NA |

**Table 4 Subgroup and sensitivity test（based on the type of stent used in different studies）.**

BMS: bare metal stent; DES: drug-eluting stent; NA: not applicable; OS: Observational study; RCT: randomized controlled trial.

*P for interaction: difference between different subgroup in the same study design
